# Mixed silage of sweet sorghum and aerial parts of licorice modulate growth, rumen function, and microbial profiles in Karakul sheep

**DOI:** 10.3389/fmicb.2026.1855333

**Published:** 2026-07-01

**Authors:** Jiao Wang, Xin Ren, Qihui Wu, Jie Wan, Mingxin Zhao, Xuewen Chen, Abdul Shakoor Chaudhry, Sujiang Zhang

**Affiliations:** 1 College of Life Science and Technology, Tarim University, Alar, China; 2 College of Animal Science and Technology, Tarim University, Alar, China; 3 Key Laboratory of Livestock and Grass Resources Utilization Around Tarim, Ministry of Agriculture and Rural Areas (Co-Construction by Ministries and Provinces), Tarim University, Alar, China; 4 School of Natural and Environmental Sciences, Newcastle University, Newcastle upon Tyne, United Kingdom

**Keywords:** bioactive, cereal silage, fermentation, growth, microbial diversity

## Abstract

To alleviate the shortage of high-quality roughage resources in arid areas, this study aimed to investigate the potential of ensiling sweet sorghum (SS) with aerial parts of licorice (APL) to support sustainable sheep production. Fifty healthy 3-month-old Karakul sheep (20.5 ± 0.87 kg) were randomly divided into five groups and fed with mixed silage diets of SS and APL in ratios of 100:0, 75:25, 50:50, 25:75 and 0:100 (dry matter basis). The measurement period lasted 60 days. The results showed that the sheep consuming 25 SS: 75 APL had the highest average daily gain and the lowest feed conversion ratio, as well as increased serum total protein, albumin, and high-density lipoprotein cholesterol. With increased APL in the diet, rumen pH, the concentration of ammonia nitrogen (NH_3_–N), the proportions of propionic acid (PA) and butyric acid (BA) linearly increased, while the acetic acid (AA) proportion and the AA/PA ratio linearly decreased. As APL increased in the diet, the Alpha diversity index of rumen bacterial community decreased, while the relative abundance of *Proteobacteria*, *Ruminococcus*, *Oscillospira,* and *Butyrivibrio* increased. The relative abundance of *Oscillospira*, *Desulfovibrio*, *Coprococcus,* and *Bacillaceae_Bacillus* was positively correlated with rumen pH, NH_3_–N concentration, the proportion of PA in rumen fluid (*p* < 0.05), and negatively correlated with the AA/PA ratio (*p* < 0.05). Microbial metabolic pathways such as amine and polyamine biosynthesis and secondary metabolite degradation were positively correlated with NH_3_–N concentration and PA proportion (*p* < 0.05). In summary, the mixed silage of SS and APL at the ratio of 25:75 effectively improved the growth performance, protein and lipid metabolism, rumen fermentation patterns, and rumen microbial community structure and functional pathways of Karakul sheep. This study provides data supporting the efficient utilization of characteristic feed resources in arid regions.

## 1 Introduction

The shortage of high-quality forage greatly limits the expansion of livestock farming in arid regions (Wang et al., 2021; Zhang et al., 2024). In an arid environment, how to effectively utilize local forage resources and build a balanced and stable feed supply system has become the key to the sustainable development of animal husbandry in the region. Therefore, integrating regionally available plant resources and developing new feed mixtures is of great practical significance for sustainable sheep production.

Sweet sorghum (*Sorghum bicolor,* SS) as a drought-tolerant and high-yielding feed crop that is promoted locally, has become an important forage material for ensiling due to its high biomass and fermentable carbohydrate content (Malabadi et al., 2022; Zhang et al., 2016). However, single SS silage is limited as a high-quality forage, such as low protein content and unbalanced nutritional structure, which limit its full application in efficient fattening of ruminants (Reddy and Blümmel, 2021; Wang et al., 2021). Licorice (*Glycyrrhiza uralensis*) is a perennial leguminous plant recognized for its tolerance to drought and saline-alkali stress, with natural distribution spanning Central Asia, Mongolia, Russia and China (Kondo et al., 2007). Its commercial value has traditionally derived from root glycyrrhizin extraction, while the aerial parts of licorice (APL), comprising stems, leaves, and inflorescences are typically discarded as agricultural by-products, representing a significant loss of biomass (Jiang L. et al., 2021). Recent studies have confirmed that APL contains moderate levels of crude protein (12–16% DM), structural carbohydrates, and a range of secondary metabolites including flavonoids and triterpenoid saponins (Jiang L. et al., 2021; Dang et al., 2024). Utilizing APL as a feed resource could not only reduce agricultural waste but also provide a roughage that supplements protein and minerals for animal production.

Mixing SS with APL for silage is expected to achieve nutritional and functional complementarity. SS provides sufficient fermentable carbohydrates to support lactic acid fermentation, while APL contributes crude protein and may introduce secondary metabolites that could modulate rumen microbial communities and fermentation patterns. Chen et al. (2024) reported that SS:APL (50:50) silage exhibited improved fermentation characteristics and *in vitro* digestibility compared to APL silage alone, indicating synergistic effects during ensiling.

We hypothesized that incorporating APL into SS-based silage would exert a dual effect: (1) nutritionally, by elevating dietary crude protein and reducing fiber content, thereby improving nitrogen supply and intake potential; and (2) functionally, by introducing plant secondary metabolites that may favorably shift rumen fermentation toward propionate and butyrate production and enrich specific bacterial taxa associated with protein metabolism and energy harvesting. This hypothesis was tested by feeding Karakul sheep with total mixed rations containing SS-APL mixed silages at five inclusion ratios and evaluating growth performance, serum metabolites, rumen fermentation parameters, and rumen bacterial community structure and predicted metabolic function.

## 2 Materials and methods

### 2.1 Preparation of silage

SS was planted in April 2023 and harvested at the milk stage at the end of September whereas the APL were purchased from local companies and harvested at the same time. The two forages were crushed to 2–3 cm in size by a straw chopper (9RSJ-6, Leling City He Yi Machinery Equipment Co., Ltd., Shandong, China) and processed according to the dry matter ratios (100:0, 75:25, 50:50, 25:75, 0:100) of SS to APL. After mixing evenly, the silage bags were produced using a round baling and wrapping machine (9YDB-0.5, Leling City He Yi Machinery Equipment Co., Ltd., Shandong, China). Each bale was approximately 0.6 m in diameter and weighed about 50 kg, compacted and wrapped with six layers of stretch film to ensure airtight conditions. The bales were stored at room temperature (approximately 25 °C) for 60 days. At the time of feeding, the silage appeared yellowish-green with a pleasant acidic aroma and showed no signs of mold or spoilage. The pH was approximately 4.0 and the dry matter content was about 35%, indicating good silage quality. The chemical composition and bioactive component contents of the five mixed silages are presented in Supplementary Table S1.

### 2.2 Experimental design and experimental diets

The trial was approved by the Animal Ethics Committee of Tarim University (No. 2023022). Fifty 3-month-old healthy Karakul sheep, weighing (20.5 ± 0.87 kg), were randomly divided into five groups of ten sheep per group. Each group was fed a total mixed diet with a 50:50 ratio of concentrate to forage, which was calculated based on the nutrient requirements for growing meat sheep (NY/T 816-2021). The ingredient and nutrient compositions of the experimental diets are shown in Table 1.

**Table 1:** Ingredient and nutrient composition of experimental diets (DM basis).

Items	100%SS^a^	75%SS	50%SS	25%SS	0%SS
Ingredients, %					
Silage mixture	30.00	30.00	30.00	30.00	30.00
Wheat straw	20.00	20.00	20.00	20.00	20.00
Corn	15.00	15.00	15.00	15.00	15.00
Wheat bran	23.00	23.00	23.00	23.00	23.00
Soybean meal	5.00	5.00	5.00	5.00	5.00
Limestone	0.30	0.30	0.30	0.30	0.30
NaCl	0.70	0.70	0.70	0.70	0.70
NaHCO_3_	1.00	1.00	1.00	1.00	1.00
Premix^b^	5.00	5.00	5.00	5.00	5.00
Total	100.00	100.00	100.00	100.00	100.00
Nutrients^c^, %					
ME, (MJ/kg)	12.53	12.58	12.79	12.96	13.05
CP	13.38	14.06	14.81	15.65	16.05
NDF	62.34	58.62	56.40	55.41	53.19
ADF	20.13	19.79	18.33	17.07	16.42
EE	3.51	4.18	4.23	4.31	4.35
Ash	11.36	13.17	13.09	14.28	14.33
Ca	1.11	1.16	1.22	1.26	1.29
P	0.44	0.45	0.45	0.45	0.45

a SS, sweet sorghum.

b One kg of premix provided the following: VA 150000 IU, VD_3_ 40,000 IU, VE 750 IU, Se 10 mg, I 10 mg, Cu 200 ~ 700 mg, Fe 1,000 mg, Mn 200 mg, Zn 1,000 ~ 3,000 mg, Ca 80 ~ 200 g, NaCl 80 ~ 240 g.

c ME, metabolic energy; CP, crude protein; NDF, neutral detergent fiber; ADF, acid detergent fiber; EE, ether extract. Nutrient levels were measured values except ME. ME was calculated according to Tables of Feed Composition and Nutritive Values in China (2022) and Nutrient Requirements of Meat Sheep (NY/T 816–2021). ME = 0.82 × (TDN/4.4).

### 2.3 Feeding and management

The test sheep were housed in individual stalls bedded with sawdust and had free access to feed and water ad libitum. The feed was offered twice daily, firstly at 09:00 and secondly at 19:00. The feed refusals were recorded and the amount of the next feed offered was adjusted accordingly to allow about 10% refusals. The pre-trial adaptation period was 15 days and the formal trial period for recording observations was 60 days. Regular hygiene measures were followed to disinfect and clean the housing environment during the trial.

### 2.4 Growth performance

Each sheep was weighed for initial (IBW) and final body weight (FBW) before the morning feeding on day 1 and day 60, respectively. Average dry matter feed intake (ADMI) per sheep was calculated based on the amount of feed offered minus the amount of feed refused, both on a dry matter basis. The average daily gain (ADG), the ADMI and feed conversion ratio (FCR) were calculated as follows:


ADG=(FBW−IBW)/Test days;

ADMI=Totaldrymatter intake/Test days;

FCR=ADMI/ADG.

### 2.5 Determination of serum biochemical indicators

On the 60th day of the trial period, before morning feeding, 5 mL of blood was collected through the jugular vein using a non-anticoagulant blood collection tube (KWS, Hebei Kangweishi Medical Technology Co., Ltd., Shijiazhuang, China), tilted and left to stand for 20 min, then centrifuged at 1,000 × *g* for 15 min. The supernatant was collected and stored at −80 °C for the determination of serum biochemical indicators by using a fully automatic biochemical analyzer (BK-280, Shandong Boke Bioindustry Co., Ltd., Shandong). The serum samples were analysed for total protein (TP), albumin (ALB), globulin (GLB), triglycerides (TG), total cholesterol (TC), high-density lipoprotein (HDL), low-density lipoprotein (LDL), urea nitrogen (BUN), glucose (GLU), alanine aminotransferase (ALT) and aspartate aminotransferase (AST) by using relevant protocols (Song et al., 2018).

### 2.6 Determination of rumen fermentation parameters

Rumen fluid (RF) samples were obtained at the end of the trial from 30 sheep (*n* = 6 per group) post-slaughter. For each animal, the rumen contents were homogenized and strained through four layers of sterile cheesecloth. The resulting filtrate was promptly aliquoted into 50 mL centrifuge tubes. The pH of the RF was immediately measured using a portable pH meter (HI8424, Beijing Hanna Instruments Science and Technology Co. Ltd., Beijing, China). Subsequently, all samples were transferred in liquid nitrogen tanks, then moved to a −80 °C freezer for the determination of rumen fermentation parameters and microbial diversity.

The ammonia-nitrogen (NH_3_–N) content was analyzed by the phenol-hypochlorite reaction according to the steps outlined by Weatherburn (1967). Standard stock solutions of acetic acid (AA), propionic acid (PA), butyric acid (BA), isobutyric acid (IBA), valeric acid (VA), and isovaleric acid (IVA) were prepared (García-Villalba et al., 2012). For sample analysis, each RF sample was first diluted twofold with deionized water. An aliquot of 200 μL of the diluted sample was then mixed with 50 μL of 15% (v/v) phosphoric acid, 10 μL of 75 μg/mL internal standard solution (isocaproic acid), and 140 μL of diethyl ether, followed by vortex homogenization for 1 min. After centrifugation of the acidified samples at 4 °C and 12,000 rpm for 10 min, the supernatant was collected and analyzed for volatile fatty acids (VFAs) as described by Zhang et al. (2019) and Hsu et al. (2019).

### 2.7 Analysis of rumen microbiota diversity

Microbial genomic DNA was extracted using the OMEGA Soil DNA Kit (D5635-02) (Omega Bio-Tek, Norcross, GA, USA) kit. The extracted DNA was subjected to 0.8% agarose gel electrophoresis for molecular size determination, and the DNA was quantified using Nanodrop (NC2000, Thermo Scientific, USA). The PCR amplification selected bacterial 16S rRNA V3-V4 region-specific primers, 338F (5′-ACTCCTACGGGAGGCAGCA-3′), 806R (5′-GGACTACHVGGGTWTCTAAT-3′) were used to perform PCR using NEB Q5 DNA high-fidelity polymerase. The PCR amplification protocol was carried out as described by Wang et al. (2022).

The library construction was carried out using Illumina’s TruSeq Nano DNA LT Library Prep Kit. For qualified libraries, double-ended sequencing was performed on the Illlumina MiSeq instrument. Sequence denoising or operational taxonomic unit (OTU) clustering was performed following the QIIME2 dada2 analysis process or the analysis process of Vsearch (v2.13.4_linux_x86_64) software to show the specific composition of each sample (group) at the phylum and genus levels. Alpha diversity indices were calculated to evaluate the microbial richness and evenness within each sample. Beta diversity was assessed using principal coordinate analysis (PCoA) based on Bray–Curtis, unweighted UniFrac, and weighted UniFrac distance matrices. Permutational multivariate analysis of variance (PERMANOVA) with 999 permutations was performed to test the significance of differences in rumen bacterial community composition among the five dietary groups. To identify differentially abundant genera, LEfSe analysis was performed with an LDA threshold of 2.0. The Wilcoxon rank-sum test and Benjamini–Hochberg false discovery rate (FDR) correction were applied, and an FDR-adjusted *p*-value <0.05 was considered statistically significant. Based on the results of 16S rRNA gene sequencing, the microbiota metabolic functions of the samples were predicted and the differential pathways were identified. Finally, the Spearman rank correlation analysis method was used to analyze the relationship between rumen microbiota and metabolic pathways and rumen fermentation parameters.

### 2.8 Statistical analysis

The experimental data were collated using Excel 2019 and analyzed using SPSS 26.0 software (IBM Corp., Armonk, NY, USA). Serum biochemical indicators and rumen fermentation parameters were analyzed by one-way analysis of variance. When a significant treatment effect was observed, Duncan’s multiple range test was used for post-hoc comparisons. The results are presented as the mean and standard error of the mean (SEM). A *p*-value <0.05 was considered to declare differences between means to be statistically significant. Furthermore, linear and quadratic regression analyses were conducted to examine the dose–response relationships between the different mixing ratios of SS and APL and the variables of growth performance, serum biochemistry, and rumen fermentation parameters.

## 3 Results

### 3.1 Growth performance

According to Table 2, there was no significant difference in IBW among the groups of Karakul sheep (*p* > 0.05). With the increase in the proportion of APL in the mixed silage, the FBW and ADG of Karakul sheep exhibited a significant quadratic response (*p* < 0.05), characterized by an initial increase followed by a decrease. Specifically, the FBW of the 25%SS group was significantly higher than that of the 100%SS and 75%SS groups (*p* < 0.05), and the ADG of the 50%SS and 25%SS groups was significantly higher than that of the 100%SS and 75%SS groups (*p* < 0.05). There was no significant difference in ADMI among the groups (*p* > 0.05). With the increase in the proportion of APL in the mixed silage, FCR showed a secondary trend of first decreasing and then increasing (*p* < 0.05), among which the 25%SS group had the lowest FCR, significantly lower than the 100%SS group and the 75%SS group (*p* < 0.05).

**Table 2:** Effects of mixed silages of sweet sorghum and aerial parts of licorice on growth performance of Karakul sheep.

Items^1^	Groups					SEM	*p*-value		
	100%SS	75%SS	50%SS	25%SS	0%SS		Treatment	Linear	Quadratic
IBW, kg	20.47	20.65	20.14	20.23	20.83	0.386	0.371	0.725	0.167
FBW, kg	29.48^c^	30.91^b^	32.45^ab^	33.37^a^	31.84^ab^	0.422	<0.001	<0.001	0.031
ADMI, g/d	924.78	969.81	1000.28	1018.67	970.14	24.105	0.091	0.071	0.029
ADG, g/d	150.31^d^	174.13^c^	199.91^ab^	215.20^a^	189.71^bc^	7.030	<0.001	<0.001	<0.001
FCR	6.24^a^	5.61^ab^	5.02^bc^	4.77^c^	5.26^bc^	0.240	0.001	0.001	0.015

1 SS, sweet sorghum; SEM, standard error of the mean; IBW, initial body weight; FBW, final body weight; ADMI, average dry matter intake; ADG, average daily gain; FCR, feed conversion ratio. In the same row, values with the same letter or no letter superscripts mean no significant difference (*p* > 0.05), while values with different letter superscripts mean significant difference (*p* < 0.05).

### 3.2 Serum biochemical indicators

As shown in Table 3, feeding Karakul sheep with mixed silage of SS and APL had no significant effect on serum ALB, GLB, AST, TC, LDL, and GLU levels (*p* > 0.05). The TP content showed significant differences among the groups (*p* < 0.05), with the 25% SS group having the highest level, which exhibited a linear increasing trend with the rise in APL ratio (*p* < 0.001). The HDL content also demonstrated a linear increasing trend with the increase in APL ratio (*p* < 0.01), and the 100% SS group was significantly lower than the other four groups (*p* < 0.05).

**Table 3:** Effects of mixed silage of sweet sorghum and aerial parts of licorice on serum biochemical indicators of Karakul sheep.

Items^1^	Groups					SEM	*p*-value		
	100%SS	75%SS	50%SS	25%SS	0%SS		Treatment	Linear	Quadratic
TP, g/L	62.44^c^	62.45^c^	64.27^bc^	66.72^a^	66.37^ab^	1.00	0.003	<0.001	0.974
ALB, g/L	24.51	24.73	25.48	26.61	25.74	2.69	0.935	0.486	0.804
GLB, g/L	37.93	37.72	38.78	40.11	40.63	2.96	0.816	0.267	0.829
ALT, U/L	18.86	18.85	16.18	14.46	14.30	2.14	0.142	0.018	0.911
AST, U/L	103.74	101.70	96.32	93.08	100.88	8.50	0.723	0.203	0.763
TG, mmol/L	0.29	0.26	0.24	0.21	0.21	0.04	0.178	0.023	0.569
TC, mmol/L	0.87	0.97	1.04	1.06	1.07	0.14	0.606	0.149	0.559
HDL, mmol/L	0.70^b^	0.83^a^	0.85^a^	0.85^a^	0.90^a^	0.04	0.005	0.001	0.112
LDL, mmol/L	0.22	0.25	0.27	0.28	0.30	0.05	0.565	0.113	0.879
BUN, mmol/L	4.00	4.06	4.15	4.79	5.68	0.82	0.268	0.049	0.330
GLU, mmol/L	3.83	3.80	3.72	3.70	3.47	0.53	0.961	0.499	0.813

1 SS, sweet sorghum; SEM, standard error of the mean; TP, total protein; ALB, albumin; GLB, globulin; TG, triglycerides; TC, total cholesterol; HDL, high-density lipoprotein; LDL, low-density lipoprotein; BUN, blood urea nitrogen; GLU, glucose; ALT, alanine aminotransferase; AST, aspartate aminotransferase. In the same row, values with the same letter or no letter superscripts mean no significant difference (*p* > 0.05), while with different letter superscripts mean significant difference (*p* < 0.05).

### 3.3 Rumen fermentation parameters

According to Table 4, with the increase in the proportion of APL in mixed silage, the pH value and NH_3_–N concentration of RF in Karakul sheep showed a very significant linear upward trend (*p* < 0.05), while the pH value of RF in 100%SS group, 25%SS group and 0%SS group increased significantly (*p* < 0.05). The concentration of ammonia nitrogen in the rumen of the 25%SS and 0%SS groups was significantly higher than that of the other three groups (*p* < 0.05). There was no significant difference (*p* > 0.05) in VFAs, VA, IVA, IBA in the RF of different groups of sheep. The content of AA in the RF of 100%SS and 75%SS was significantly higher than that of 25%SS and 0%SS (*p* < 0.05), and the content of PA in the RF of 0%SS was significantly higher than that of the first three groups (*p* < 0.05). The rumen VA content showed a quadratic trend (first increasing and then decreasing) as the proportion of APL in the mixed silage increased (*p* < 0.05), the VA content in the rumen of the 25% group was significantly higher than that of the first three groups (*p* < 0.05), and the AA/PA in the rumen of the 25%SS and 0%SS groups was significantly lower than that of the 100%SS group (*p* < 0.05).

**Table 4:** Effects of mixed silage of sweet sorghum and aerial parts of licorice on rumen fermentation parameters of Karakul sheep.

Items^1^	Groups					SEM	*P*-value		
	100%SS	75%SS	50%SS	25%SS	0%SS		Treatment	Linear	Quadratic
pH	6.67^c^	6.73^bc^	6.77^bc^	6.87^ab^	6.97^a^	0.08	0.025	0.002	0.537
NH_3_–N, mg/100 mL	9.53^c^	10.18^c^	11.84^b^	13.62^a^	14.50^a^	0.41	<0.001	<0.001	0.605
VFAs	74.87	75.33	75.90	76.07	77.28	4.44	0.985	0.587	0.927
AA, %	63.21^a^	61.17^ab^	60.07^bc^	57.90^cd^	56.32^d^	1.34	0.003	<0.001	0.968
PA, %	23.89^b^	24.61^b^	24.87^b^	26.20^ab^	27.64^a^	1.00	0.026	0.002	0.365
BA, %	9.31^d^	10.19^c^	11.02^b^	11.84^a^	11.62^ab^	0.35	<0.001	<0.001	0.037
VA, %	0.90	1.01	1.01	1.06	1.01	0.11	0.671	0.286	0.364
IVA, %	1.67	1.87	1.86	1.85	2.02	0.15	0.307	0.069	0.864
IBA, %	1.03	1.16	1.17	1.16	1.39	0.18	0.462	0.113	0.738
AA/PA	2.65^a^	2.51^ab^	2.42^ab^	2.21^bc^	2.04^c^	0.15	0.018	0.001	0.642

1 SS, sweet sorghum; SEM, standard error of the mean; VFAs, volatile fatty acids; AA, acetic acid; PA, propionic acid; BA, butyric acid; VA, valeric acid; IVA, isovaleric acid; IBA, isobutyric acid. In the same row, values with the same letter or no letter superscripts mean no significant difference (*p* > 0.05), while with different letter superscripts mean significant difference (*p* < 0.05).

### 3.4 Rumen microbial communities

**Figure 1:**
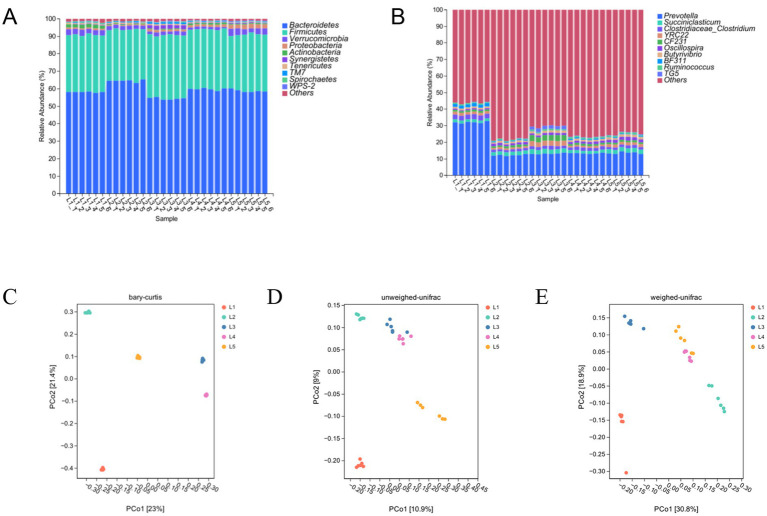
Overall structure and intergroup differences of the rumen bacterial community. **(A)** Phylum-level composition; **(B)** Genus-level composition; **(C)** Principal coordinate analysis (PCoA) based on Bray-Curtis distance matrix; **(D)** PCoA based on unweighted Unifrac distance; **(E)** PCoA based on weighted Unifrac distance. L represents rumen fluid; groups 1, 2, 3, 4, and 5 represent sweet sorghum to aerial parts of licorice ratios of 100:0 (100%SS), 75:25 (75%SS), 50:50 (50%SS), 25:75 (25%SS), and 0:100 (0%SS), respectively.

The most abundant phyla in each group were *Bacteroidetes* and *Firmicutes* (Figure 1A). The 75%SS group had the highest relative abundance of *Bacteroidetes* (64.61%). The 50%SS group had the highest relative abundance of *Firmicutes* (36.22%), *Synergistetes* (2.10%), and *Tenericutes* (0.72%), and these values were numerically higher than those in the other four groups. The 0%SS, 25%SS, and 50%SS groups had higher relative abundance of *Proteobacteria* (2.41, 1.31, and 1.06%, respectively) than the 100%SS (0.83%) and 75%SS (0.57%) groups.

At the genus level (Figure 1B), the relative abundance of *Prevotella* was higher in the 100%SS group (32.11%) than in the other four groups. The relative abundance of *Ruminococcus* was numerically higher in the 0%SS (0.77%), 25%SS (0.83%), and 50%SS (0.77%) groups than in the 100%SS (0.66%) and 75%SS (0.55%) groups. In the 0%SS group, *Butyrivibrio* (1.51%) and *Oscillospira* (1.82%) were also numerically higher than in the other four groups.

PERMANOVA confirmed that the mixing ratio significantly affected the rumen bacterial community structure (Figures 1B–C, Bray-Curtis: *R*^2^ = 0.842, *p* = 0.001; unweighted UniFrac: *R*^2^ = 0.354, *p* = 0.001; weighted UniFrac: *R*^2^ = 0.925, *p* = 0.001).

**Figure 2:**
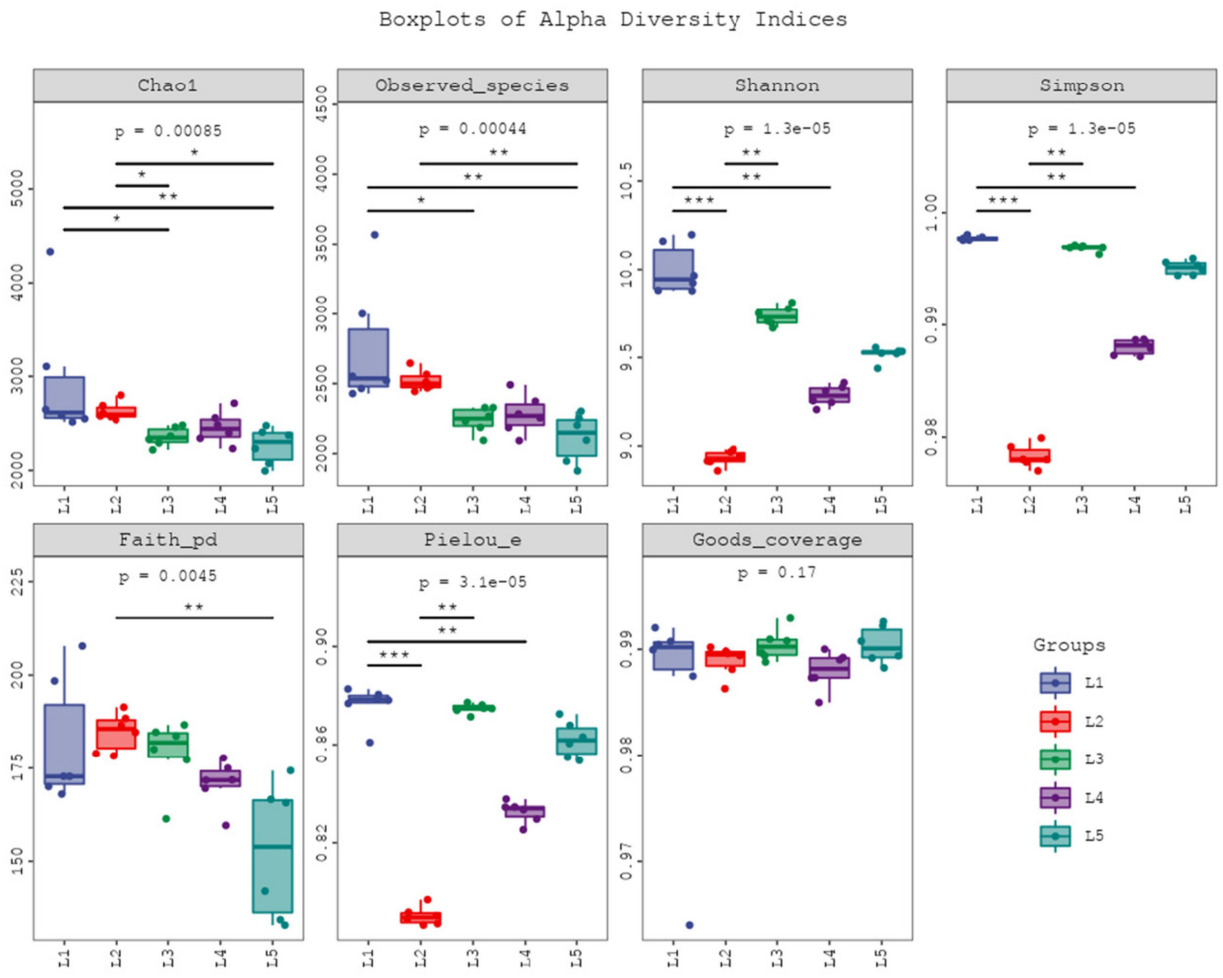
Alpha diversity index. L represents rumen fluid, and 1, 2, 3, 4, 5 represent sweet sorghum to aerial parts of licorice ratios of 100:0 (100%SS), 75:25 (75%SS), 50:50 (50%SS), 25:75 (25%SS), and 0:100 (0%SS), respectively. *indicates *p* < 0.05, **indicates *p* < 0.01.

As shown in Figure 2, Chao1 and Observed-species indices decreased significantly with the reduction of SS proportion (*p* < 0.001), and the 100%SS group had markedly higher species richness than other groups. The Shannon and Simpson indices peaked in the 100%SS group, with highly significant differences among all groups (*p* < 0.001). The 75%SS group presented the lowest Pielou’s evenness index, which was significantly lower than that of the other groups (*p* < 0.001). The Faith’s-pd index revealed that the 0%SS group had significantly lower phylogenetic diversity than the 100%SS group (*p* < 0.01). The Good’s-coverage index was above 0.97 in all groups with no significant intergroup difference (*p* = 0.17), indicating adequate sequencing depth.

**Figure 3:**
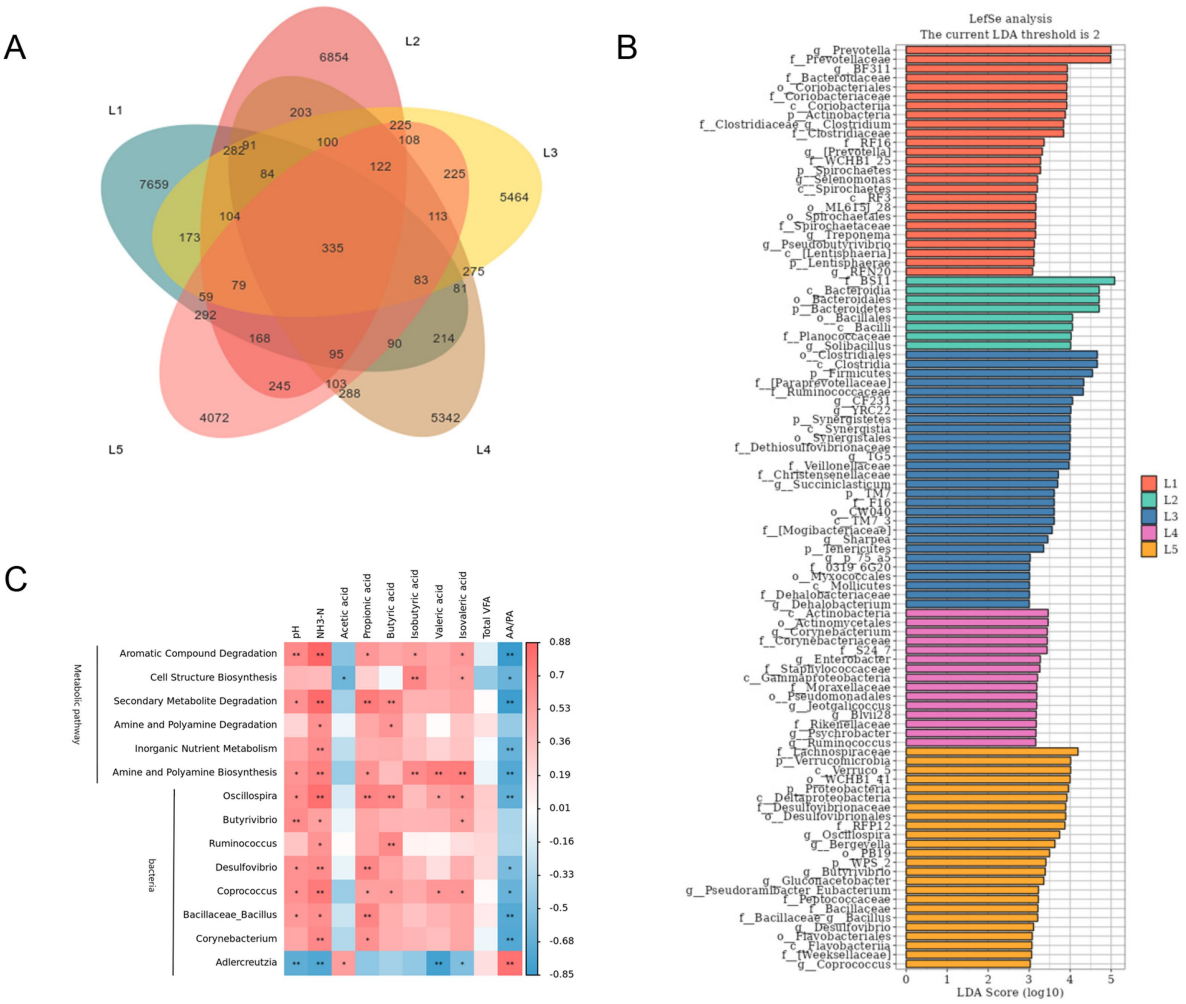
Rumen bacterial community structure, differentially abundant genera, and their correlations with fermentation parameters. **(A)** Venn diagram showing the number of shared and unique OTUs across the five treatment groups. **(B)** LEfSe analysis (LDA > 2.0, FDR-adjusted *p* < 0.05) identifying differentially abundant genera among the five groups. **(C)** Spearman correlation analysis between rumen microbiota/metabolic pathways and rumen fermentation parameters (*p < 0.05, **p < 0.01). L represents rumen fluid; groups 1, 2, 3, 4, and 5 represent sweet sorghum to aerial parts of licorice ratios of 100:0 (100%SS), 75:25 (75%SS), 50:50 (50%SS), 25:75 (25%SS), and 0:100 (0%SS), respectively.

The Venn diagram (Figure 3A) was constructed using the OTU abundance table. Each circle in the figure represents a group of samples, and the overlapping regions between circles indicate the number of shared OTUs. The Karakul sheep were fed with mixed silage in the ratios of 100:0, 75:25, 50:50, 25:75, and 0:100 (SS to APL). The groups had 7,994, 7,189, 5,799, 5,677, and 4,407 OTUs, respectively, with 7,659, 6,854, 5,464, 5,342, and 4,072 unique OTUs. As the proportion of SS in the mixed silage decreased, the number of OTUs in the rumen showed a declining trend.

LefSe analysis identified significantly differential rumen microbial biomarkers (LDA score > 2) across all groups (Figure 3B). The 100%SS group was characterized by the genera *Prevotella*, *Clostridium*, *Treponema*, and *Pseudobutyrivibrio*. The 25%SS group was distinguished by the enrichment of *Corynebacterium*, *Enterobacter* and *Ruminococcus*. Meanwhile, *Coprococcus*, *Butyrivibrio*, *Oscillospira*, and *Bacillus* were identified as the signature genera for the 0%SS group, serving as potential biomarkers to differentiate the treatments.

Rumen microbial metabolic pathway prediction analysis is shown in Table 5. At the primary functional category level (Level 1), microbial metabolic activities were predominantly concentrated in “Degradation/Utilization/Assimilation” and “Biosynthesis.” Further analysis of specific metabolic pathways (Level 2) revealed that, with the increasing proportion of APL in the diet, significant changes occurred in certain metabolic pathways. Compared with the 100%SS and 75%SS groups, the activities of inorganic nutrient metabolism, amine and polyamine degradation, secondary metabolite degradation, and aromatic compound degradation pathways were significantly higher in the 25%SS and 0%SS groups (*p* < 0.05). The activity of the amino acid degradation pathway in the 100%SS group was significantly lower than that in all other groups (*p* < 0.05). Among the biosynthesis pathways, the activity of amine and polyamine biosynthesis in the 0%SS group was significantly higher than that in the 100%SS group, and its secondary metabolite biosynthesis activity was also significantly higher than that in the first four groups (*p* < 0.05). Additionally, the activity of carbohydrate biosynthesis in the 25%SS group was significantly higher than that in the first three groups (*p* < 0.05).

**Table 5:** Analysis of rumen microbial metabolic pathways.

Items^1^	Groups					SEM	*p*-value		
	100%SS	75%SS	50%SS	25%SS	0%SS		Treatment	Linear	Quadratic
Degradation/utilization/assimilation									
Inorganic nutrient metabolism	8.17^b^	8.33^b^	7.83^b^	9.00^a^	9.00^a^	0.45	0.054	0.028	0.270
Amine and polyamine degradation	7.17^b^	6.67^b^	8.00^a^	8.17^a^	8.00^a^	0.53	0.036	0.013	0.724
Secondary metabolite degradation	12.33^b^	12.00^b^	12.50^ab^	13.00^a^	13.00^a^	0.30	0.010	0.002	0.412
Amino acid degradation	11.50^b^	14.33^a^	14.00^a^	13.67^a^	14.17^a^	0.74	0.004	0.009	0.025
Aromatic compound degradation	16.83^c^	17.67^c^	20.00^bc^	25.17^a^	23.67^a^	2.08	0.001	<0.001	0.742
Biosynthesis									
Amine and polyamine biosynthesis	4.00^b^	4.50^ab^	4.50^ab^	4.83^a^	5.00^a^	0.32	0.047	0.003	0.700
Secondary metabolite biosynthesis	10.00^b^	9.83^b^	9.67^b^	9.50^b^	11.50^a^	0.56	0.010	0.042	0.007
Carbohydrate biosynthesis	20.00^b^	19.00^b^	19.00^b^	21.33^a^	20.33^ab^	0.62	0.004	0.040	0.166

1 SS, sweet sorghum; SEM, standard error of the mean. In the same row, values with the same letter or no letter superscripts mean no significant difference (*p* > 0.05), while with different letter superscripts mean significant difference (*p* < 0.05).

### 3.5 Correlation analysis

Correlation analysis of rumen fermentation parameters with metabolic pathways is shown in Figure 3C. The aromatic compound degradation, secondary metabolite degradation, amine and polyamine biosynthesis were positively correlated with pH, NH_3_–N concentration, PA (*p* < 0.05), and negatively correlated with AA/PA (*p* < 0.05); amine and polyamine biosynthesis was positively correlated with IBA, VA and IVA (*p* < 0.05).

The correlation analysis between rumen fermentation parameters and rumen microbial community is as follows: *Oscillospira*, *Butyrivibrio*, *Ruminococcus*, *Coprococcus*, and *Bacillaceae Bacillus* were positively correlated with the RF pH and NH_3_–N concentration (*p* < 0.05). The *Oscillospira*, *Desulfovibrio*, *Coprococcus* were positively correlated with the RF PA and BA (*p* < 0.05). *Oscillospira* is positively correlated with the RF IBA, VA, and IVA (*p* < 0.01), and negatively correlated with AA/PA (*p* < 0.01). On the contrary, *Adlercreutzia* was negatively correlated with the NH_3_–N concentration, PA, BA, IBA, VA, and IVA content in RF (*p* < 0.05), and positively correlated with AA and AA/PA (*p* < 0.05).

## 4 Discussion

### 4.1 Growth performance

As the proportion of APL in the mixed silages increased, dietary CP and energy levels rose, while NDF and ADF contents declined. Higher CP content generally promotes growth performance in ruminants, as it is positively correlated with body weight, ADG, and ADMI (Zhang et al., 2017; Wang X. et al., 2020). In this study, ADG was higher in the 50%SS, 25%SS, and 0%SS groups compared to the 100%SS and 75%SS groups. However, ADG in the 0%SS group was lower than in the 50%SS and 25%SS groups. This may be attributed to impaired silage quality resulting from excessively high CP content, as previously reported in the same silage materials (Chen et al., 2024), where 0%SS silage showed elevated pH and NH_3_–N and reduced lactic acid. Poor silage quality is known to reduce palatability and nutrient digestibility (Kung et al., 2018), which likely limited ADG in the 0%SS group. Concurrently, the reduction in NDF and ADF content with higher APL proportion likely contributed to a slight increase in ADMI, as fiber content is negatively correlated with feed intake (Arelovich et al., 2008; Chen et al., 2021). Moreover, APL contains bioactive medicinal components, and its inclusion in animal diets has been shown to enhance growth performance. Studies report that herbal additives, including flavonoids from licorice, can significantly improve ADG, ADMI, and FCR in sheep (Qi et al., 2017; Redoy et al., 2020; Chen et al., 2023). Thus, both nutritional and bioactive factors in APL may collectively explain the improved growth performance observed in sheep fed higher proportions of APL.

### 4.2 Serum biochemical indicators

Serum biochemical indicators reflect the overall physiological and nutritional status of animals. This experiment investigated the effects of different proportions of mixed silage containing SS and APL on the metabolism and health of Karakul sheep. The TP and ALB, synthesized primarily in the liver, are key indicators of protein absorption, anabolism, and overall nutritional status (Cerón et al., 2005). Their synthesis is influenced by dietary protein intake (Connell et al., 1997). In this study, both TP and ALB levels increased with the proportion of APL in the diet. By day 60, ALB was significantly higher in the 25 and 0% SS groups than in the 75 and 100% SS groups, suggesting that APL provided better and more readily absorbed protein, thereby improving protein synthesis and nutritional status-a finding consistent with Yin et al. (2025). BUN is the end product of protein metabolism, reflects protein utilization efficiency and amino acid balance (Mahmood et al., 2024). The BUN did not differ among groups, indicating improved nitrogen retention and reduced muscle protein catabolism (Van Bibber-Krueger et al., 2015), which suggests better protein utilization and reduced renal excretory load. The ALT and AST are liver enzymes released into blood upon hepatocyte damage. Their activities remained within normal physiological ranges across all groups, indicating no adverse effect of any diet on liver function. Increasing APL proportion significantly raised HDL and tended to reduce TG. Similar lipid-modulating effects have been reported in yaks fed licorice (Jiang et al., 2022). Saponins in licorice-also present in APL-can reduce fat accumulation via AMPK signaling (Cheng et al., 2020). The increased contents of liquiritin, glycyrrhizic acid, total flavonoids, and total phenols in diets with higher APL proportion (Supplementary Table S1) may partially contribute to these observed improvements in protein and lipid metabolism. In summary, mixed silage with an appropriate proportion of APL can enhance protein and lipid metabolism, promoting the health of Karakul sheep. The mechanism by which APL bioactive components influence lipid metabolism warrants further investigation.

### 4.3 Rumen fermentation parameters

Rumen pH, a key indicator of fermentation intensity, remained within the normal range (6.67–6.97) across all groups. Although pH decreased significantly in sheep fed higher proportions of SS, it did not fall below 6.0, indicating no severe inhibition of cellulolytic bacteria, consistent with Zhang et al. (2023). Thus, the mixed silages supported a stable rumen environment conducive to fiber digestion. The linear increase in pH with higher APL inclusion may also reflect an enhanced buffering capacity from the accumulation of basic nitrogenous compounds resulting from intensified protein degradation (Belanche et al., 2021). NH_3_–N concentration reflects microbial protein degradation and nitrogen utilization. In this study, NH_3_–N increased linearly with higher APL inclusion. This rise may be attributed to the higher crude protein content in APL (Chen et al., 2024) and the potential of licorice flavonoids to stimulate microbial protease activity, enhancing protein deamination (Zhang et al., 2020). These findings align with reports that high-protein diets elevate rumen NH_3_–N (Xia et al., 2018). As the main energy source for ruminants, VFAs profiles shifted with dietary composition. The proportion of acetic acid (AA) decreased as APL increased, likely because SS provides more fermentable carbohydrates (Zhang et al., 2015, 2024) and supports protozoal growth, a major contributor to AA production (Antonius et al., 2023). Conversely, PA and BA proportions increased with APL inclusion, consistent with studies on saponin-rich supplements (An et al., 2023; Jiang C. et al., 2021). This shift toward propionate and butyrate is energetically favorable for the host, as propionate is a more efficient precursor for hepatic gluconeogenesis compared to acetate (Ahmad et al., 2020).

### 4.4 Rumen microbial communities

Consistent with previous studies (Shao et al., 2023; Wang Q. et al., 2020), dietary composition significantly shaped the rumen microbial community in this study. In our study, it was found that an increase in the proportion of APL in mixed silage had a negative impact on the alpha diversity index, suggesting that an increase in protein levels leads to a decrease in bacterial diversity. Zhang et al. (2021) also demonstrated that a high protein level diet reduced the richness of rumen bacteria in yaks. This reduction in diversity may reflect a selective enrichment of specialized functional guilds adapted to protein-rich environments, rather than a detriment to ecosystem function (Weimer, 2015).

By analyzing the community composition at the phylum level, the most abundant phyla were *Bacteroidetes* and *Firmicutes*, which are known to play important roles in rumen fermentation (Bi et al., 2018; Hou et al., 2025; Wang et al., 2022). *Bacteroidetes* degrades cellulose and hemicellulose, directly affecting roughage utilization efficiency (Moraïs and Mizrahi, 2019). *Proteobacteria* increased significantly with higher APL inclusion. This phylum is involved in nitrogen metabolism; some members can hydrolyze urea into ammonia, providing a nitrogen source for other microorganisms (Xu et al., 2023). Zhang et al. (2021) similarly reported higher *Proteobacteria* abundance in yaks fed a high-protein diet, which was positively correlated with rumen ammonia concentration.

At the genus level, *Prevotella* (involved in starch and cellulose breakdown) was significantly higher in the 100%SS group, consistent with the higher starch and cellulose content of SS (Griswold et al., 1999). This aligns with the finding that low-protein, high-fiber diets selectively enrich *Prevotella* to enhance energy harvesting (Zhang et al., 2021). Although a previous study reported a positive correlation between saponin intake and *Prevotella* abundance (Wang et al., 2019), we did not observe this effect, likely due to the relatively low saponin levels in APL-containing silages (ranging from 1925.23 to 5697.38 μg/g, as shown in Supplementary Table S1), which may be below the threshold required to modulate *Prevotella* populations. *Ruminococcus* abundance was numerically higher in APL-rich groups. Beyond its proteolytic capacity (FitzGerald et al., 2015), *Ruminococcus* may contribute to propionate production via succinate synthesis. *Oscillospira* and *Butyrivibrio*, both known butyrate producers, increased with higher APL inclusion, consistent with the elevated BA proportion (Konikoff and Gophna, 2016). The PERMANOVA results further confirmed that the dietary mixing ratio significantly shaped the overall rumen bacterial community structure.

### 4.5 Correlating microbiota, pathways, and fermentation

The positive associations between butyrate-producing genera (*Oscillospira*, *Butyrivibrio*) and rumen pH/NH_3_–N suggest that the proliferation of these taxa is favored in a high-protein, moderate-pH environment, which may in turn stabilize the rumen ecosystem through butyrate-mediated epithelial health (Konikoff and Gophna, 2016). The negative correlation between *Ruminococcus* and AA/PA points to a metabolic coupling whereby *Ruminococcus* supplies succinate for propionate-producing bacteria like *Succiniclasticum*, contributing to improved feed efficiency (Van Gylswyk, 1995).

At the functional level, the enrichment of secondary metabolite degradation pathways in APL-rich diets indicates that the rumen microbiome actively biotransforms licorice-derived compounds (e.g., flavonoids, saponins). The products of this transformation may act as metabolic modulators, redirecting fermentation toward propionate and butyrate (Ramos-Morales et al., 2018). The activation of amine and polyamine biosynthesis pathways under high-APL conditions suggests an adaptive nitrogen conservation strategy. Rather than being lost as excess NH_3_–N, nitrogen from degraded proteins may be channeled into polyamines-compounds essential for bacterial stress tolerance and growth (Michael, 2016). The APL-enriched diet did not merely alter substrate availability; it restructured the rumen microbiome into a functionally specialized consortium that couples secondary metabolite degradation with enhanced propionate/butyrate production and nitrogen conservation.

## 5 Conclusion

This study comprehensively evaluated the effects of mixed silage with varying ratios of SS and APL on Karakul sheep. The 25:75 ratio (25%SS group) optimally promoted growth performance by increasing FBW and ADG while improving feed efficiency. It also enhanced protein and lipid metabolism, reflected in elevated serum TP, ALB, HDL and TC. The APL inclusion linearly raised rumen pH and NH_3_–N, improved VFAs profiles (higher PA and BA, lower AA and AA/PA), and enhanced energy utilization. Although reducing bacterial alpha diversity, this diet enriched beneficial genera linked to protein metabolism and butyrate production (e.g., *Ruminococcus*, *Butyrivibrio*). These genera correlated negatively with the AA/PA, and microbial functional analysis indicated that enhanced nitrogen and energy metabolism pathways underpinned the improved rumen function. In summary, the 25:75 mixed silage represents a nutritionally balanced and functionally regulatory feeding strategy, supporting sustainable roughage utilization and precision nutrition in meat sheep production in arid regions.

## Data Availability

The datasets generated for this study can be found in the NCBI Sequence Read Archive (SRA) under accession number PRJNA1238558.
