# Supplementary material for: Mixed silage of sweet sorghum and aerial parts of licorice modulate growth, rumen function, and microbial profiles in Karakul sheep
Source: Front Microbiol. 2026 Jul 1;17:1855333. doi: 10.3389/fmicb.2026.1855333 (PMC13369595; doi:10.3389/fmicb.2026.1855333)
Supplement: Supplementary file 1 [file Supplementary_file_1.DOCX]

**Methods**

Samples of sweet sorghum and licorice aerial parts were collected after ensiling for nutrient composition analysis. Dry matter (DM), crude protein (CP), ether extract (EE), ash, calcium (Ca), and phosphorus (P) contents were determined according to GB/T6435-2014, GB/T6432-2018, GB/T6433-2006, GB/T6438-2007, GB/T6436-2018, and GB/T6437-2018, respectively. Neutral detergent fiber (NDF) and acid detergent fiber (ADF) contents were determined following the method of Van Soest et al (1991).

For the determination of major bioactive components, liquiritin and glycyrrhizic acid were analyzed using high-performance liquid chromatography (HPLC) equipped with a UV detector. Total saponins, total flavonoids, and total phenols were determined using microplate assay methods following the protocols of commercial kits (purchased from Suzhou Comin Biotechnology Co., Ltd., Suzhou, China).

Table S1 Chemical composition of sweet sorghum and aerial parts of licorice mixed silage in different proportions

| Item^1^ | 100%SS | 75%SS | 50%SS | 25%SS | 0%SS | SEM | *P-*Value | | |
| --- | --- | --- | --- | --- | --- | --- | --- | --- | --- |
|  |  |  |  |  |  |  | Treatment | Linear | Quadratic |
| DM, % | 32.46 | 35.61 | 38.71 | 38.41 | 38.87 | 2.762 | 0.167 | 0.030 | 0.257 |
| CP, % | 7.54^d^ | 8.71^c^ | 10.65^b^ | 12.37^a^ | 12.94^a^ | 0.364 | <0.001 | <0.001 | 0.172 |
| NDF, % | 43.12^a^ | 40.63^ab^ | 38.77^bc^ | 36.13^c^ | 35.14^c^ | 1.732 | 0.006 | <0.001 | 0.639 |
| ADF, % | 23.50 | 23.07 | 22.50 | 22.04 | 21.61 | 0.859 | 0.256 | 0.031 | 0.958 |
| EE, % | 1.72^c^ | 2.64^b^ | 3.55^a^ | 3.92^a^ | 3.71^a^ | 0.400 | 0.001 | <0.001 | 0.025 |
| ash, % | 9.29 | 9.41 | 9.77 | 9.84 | 9.79 | 0.238 | 0.138 | 0.023 | 0.330 |
| Liquiritin, μg/g | 41.06^d^ | 50.72^c^ | 56.06^b^ | 57.62^b^ | 63.16^a^ | 1.732 | <0.001 | <0.001 | 0.026 |
| Glycyrrhizic acid, μg/g | 37.87^d^ | 66.61^c^ | 131.51^b^ | 134.22^b^ | 140.86^a^ | 2.654 | <0.001 | <0.001 | <0.001 |
| Total saponins, μg/g | 1925.23^d^ | 2784.14^c^ | 4699.50^b^ | 5292.64^a^ | 5697.38^a^ | 197.18 | <0.001 | <0.001 | 0.002 |
| Total flavonoids, mg/g | 11.81^b^ | 13.25^b^ | 20.24^a^ | 21.04^a^ | 22.03^a^ | 1.105 | <0.001 | <0.001 | 0.036 |
| Total phenols, mg/g | 15.05^d^ | 17.44^c^ | 19.11^bc^ | 19.75^ab^ | 21.53^a^ | 0.832 | <0.001 | <0.001 | 0.338 |

^1^ SS = Sweet sorghum, CP = Crude protein, NDF = Neutral detergent fiber, ADF = Acid detergent fiber, EE = Ether extract.

In the same row, values with no letter or the same superscript letters indicate no significant difference (*P* > 0.05), while different lowercase letters indicate a significant difference (*P* < 0.05).
